# Assessment of preeclampsia risk by use of serum ionized magnesium-based equation

**DOI:** 10.1080/0886022X.2017.1422518

**Published:** 2018-01-10

**Authors:** Chatchai Kreepala, Maethaphan Kitporntheranunt, Worrawat Sangwipasnapaporn, Warit Rungsrithananon, Krittanont Wattanavaekin

**Affiliations:** a Department of Internal Medicine, Faculty of Medicine, Srinakharinwirot University, Ongkharak, Thailand;; b Department of Obstetrics and Gynecology, Faculty of Medicine, Srinakharinwirot University, Ongkharak, Thailand;; c Chulabhorn Hospital, Bangkok, Thailand;; d Chandrubeksa Hospital, Nakornpathom, Thailand;; e Faculty of Medicine, Srinakharinwirot University, Nakornnayok Province, Thailand

**Keywords:** Ionized magnesium, preeclampsia, hypertension, pregnancy, renal function

## Abstract

**Background:** Preeclampsia is a common medical complication in pregnancy. It has been reported to be associated with decreased serum magnesium levels. However, there has not been evidence demonstrating utilization of change in magnesium for prediction of preeclampsia. The purpose of this study was to develop magnesium fraction-based equations which took other significant clinical risk factors into consideration for prediction of preeclampsia.

**Methods:** We collected serum total and ionized magnesium ionized magnesium levels from 84 pregnant women diagnosed with preeclampsia after week 20 of pregnancy. The ionized magnesium fraction was then calculated by the percentage ratio of ionized and total magnesium level.

**Results:** Sixty-four (76.19%) women had normal pregnancy and 20 (23.81%) developed preeclampsia. The ionized magnesium fraction was significantly lower in preeclampsia group (23.95 ± 4.7% vs. 26.28 ± 2.3%, *p* = .04). Additionally, lower ionized magnesium fraction (24.67%), teenage and elderly primigravida were significantly associated with preeclampsia (OR = 4.41, 95% CI: 1.46–13.40, OR = 5.47, 95% CI: 1.85–35.42 and OR = 11.11, 95% CI: 1.09–113.78, respectively). Consequently, we attempted to develop ionized magnesium fraction-based equations calculate risk scores for preeclampsia. The area of ROC for predictive accuracy of the model was 0.77 (*p* < .001) and ROC suggested that the score of 0.27 would be a threshold for screening preeclampsia with 70% sensitivity and 81% specificity.

**Conclusions:** Ionized magnesium fraction may have been appropriate for screening of preeclampsia. We suggested blood testing on total and ionized magnesium concentrations as well as calculation of ionized magnesium fraction in addition to routine antenatal care for better screening of the disease.

## Introduction

Magnesium is one of the important electrolytes in the body. It acts as a cofactor of several metabolic processes, especially the processes taking place in the neurological, reproductive, cardiovascular and immune systems [1–3]. About 25% of magnesium is in the binding form with albumin which is the major binding protein. Therefore, changes in serum albumin levels may affect both ionized and total magnesium concentrations [4,5].

Previous studies have indicated that high blood pressure may have been associated with low serum magnesium concentrations which could cause endothelial dysfunction [6]. In clinical practice, magnesium has been used in patients with severe preeclampsia so as to prevent seizure (eclampsia) during pregnancy [7,8]. It is believed that magnesium helps to inhibit calcium influx into the synaptic ending of neurons and results in cerebral vasodilatation, thereby preventing vasospasm found during an eclamptic event [9,10]. The previous reports demonstrated that an average magnesium level during pregnancy is about 1.7 mg/dL, while it is about 2.0 mg/dL in non-pregnant women [6,10]. Moreover, both levels of total and ionized magnesium decreased along the pregnancy period, especially in those who developed preeclampsia [11]. In addition, various reports have found association between magnesium deficiency and low birth weight, intrauterine growth retardation and fetal abnormalities [8,9,12–18]. Recent study has shown that the physiological decrease of magnesium during pregnancy is associated with endothelial dysfunction. Which could potentially mark as a factor that leads to preeclampsia [19]. Several literatures have demonstrated that early preeclampsia can be predicted by biomarkers such as serum placental growth factor (PIGF), soluble fms-like tyrosine kinase 1 and soluble endoglin [20–22]. Nevertheless, ionized magnesium still marks as a practical and cost-efficient predictor for preeclampsia [23]. The present study, therefore, aimed to figure out any association between serum magnesium concentrations and risk of preeclampsia. We attempted to investigate how the fraction of ionized magnesium changed during pregnancy and also figured out utilization of serum magnesium fraction together with other clinical risk factors for prediction of preeclampsia.

## Materials and methods

### Clinical data collection

This is a descriptive cohort study conducted among 93 pregnant women attending antenatal care at Her Royal Highness Princess Maha Chakri Sirindhorn Medical Center (MSMC) between 1 August 2014 and 29 February 2016. All the women voluntarily signed a consent form before participating in our study. Data were collected among pregnant women with maternal age ≥15 years and gestational age ≥20 weeks (the second half of pregnancy). Those with history or diagnosis of chronic hypertension, placenta previa, gestational diabetes (GDM) or chronic kidney disease with serum creatinine levels ≥0.8 mg/dL were excluded.

### Clinical assessment of preeclampsia

The patient’s history about their maternal age, gestational age, previous gestational history, medication reconciliation of antihypertensive agents and any other condition which may have been associated with preeclampsia such as edema or hypertension, had been reviewed. Preeclampsia has been diagnosed by the criteria of a systolic blood pressure (SBP) ≥ 140 mm Hg or a diastolic blood pressure (DBP) ≥ 90 mm Hg, on two occasions at least 4 h apart in a previously normotensive patient with positive urine protein (at least 1+ of dipstick urine protein) [17]. The gestational age was calculated by using date of last menstrual period and then confirmed by ultrasonography of fetus before the consideration for pregnancy termination.

Blood samples were collected after the second half of pregnancy (20 weeks) or at the diagnosis of preeclampsia by obstetricians. The laboratory parameters: serum creatinine and albumin levels were measured by the enzymatic method, and total magnesium levels were analyzed by the colorimetric method with use of Cobas^®^ 6000 analyzer (Roche Diagnostics, Bangkok, Thailand). Finally, ionized magnesium levels were measured with direct ion selective electrodes of a blood gas and critical care analyzer (STP pHOx Ultra^®^ analyzer, Bangkok, Thailand). The ionized magnesium fraction, calculated from the equation: (ionized magnesium/total magnesium) * 100.

### Statistical analysis

The researchers employed the SPSS statistical software package (version 23.0, SPSS Incorporated, Singapore) in data analyzes. After the analyzes had been done, continuous variables were presented in form of mean ± standard deviation (SD), and categorical variables were presented in form of percentages. The differences between the two groups were compared by use of the Student’s *T*-test and the Pearson chi square test for the continuous and categorical variables, respectively. The Spearman correlations coefficient was used for assessing correlation of selection algorithms of continuous and ordinal variables. A multivariable logistic regression analysis was performed to calculate coefficients, probability of disease and prediction scores. Area under the receiver operating characteristic curve (AUC ROC) was used in measuring diagnostic accuracy and *p* values ≤.05 were considered statistically significant.

Appropriate sample size is calculated by having the statistical power of sample size (1 − *β*) level at 0.80 and with *α* error probability at .05. Including the consideration of incidence of preeclampsia and incidence of hypomagnesaemia in preeclampsia, the calculated applicable sample size equates to 74 (in this study, *n* = 84) [12,24,25].

### Ethical statement

The protocol of patient’s participation was approved by the Human Research Ethics Board of Srinakharinwirot University (Issue #SWUEC/E-038/2557), the 1964 Helsinki declaration and the amendments.

### Informed consent

Informed consent was obtained from individual participant included in the study.

## Results

### Patients’ characteristics

Ninety-three pregnant women attending antenatal care at MSMC were included in the present study. However, nine women had been excluded because of GDM, chronic hypertension from lupus nephritis and hyperthyroidism. Of the remaining 84 pregnancies who participated in the study, 64 (76.19%) women had normal pregnancy and 20 (23.81%) women experienced preeclampsia.

The average maternal age was 28.14 ± 6.8 years and 29.50 ± 9.0 years for normal pregnancy and preeclampsia, respectively. We also categorized participants into three groups ‘normal maternal-age pregnancy (20–34 years), teenage pregnancy (<20 years) and elderly pregnancy (≥ 35 years)’. However, there was no significant association (*p* = .21) between incidence of preeclampsia and the maternal age in the teenage or elderly pregnancy groups when compared to the normal age pregnancy group. The average gestational age at enrollment was 31 ± 5.9 weeks. The gestational age at delivery was 39.0 ± 1.2 weeks and 38.0 ± 1.3 weeks for normal pregnancy and preeclampsia, respectively. The levels of baseline serum creatinine were similar in the both normal pregnancy and preeclampsia groups (0.52 ± 0.1 mg/dL vs. 0.56 ± 0.1 mg/dL, *p* = .09). However, there were significant association of low birth weight (1.9% vs. 35%, *p* < .001) and number of delivery by Cesarean section (33.3% vs. 80%, *p* < .001) in preeclampsia when compare to normal pregnancy as found in previous literature (Table 1). Some other complications like acute kidney injury, hepatitis or hemolysis, elevated liver enzymes and low platelet count (HELLP) syndrome which could be also found in patients with preeclampsia were not found in the present study.

**Table 1.** Characteristics of women with normal pregnancy and pregnancy with preeclampsia.

Clinical variables	Normal pregnancy	Pregnancy with preeclampsia	*p* value
Number (*n*)	64	20	
Maternal age (years ±SD)	28.14 ± 6.8	29.50 ± 9.0	.48
Normal maternal-age pregnancy (age 20–34 years, *n* = 56)	80.4%	19.6%	
Teenage pregnancy (age ≤19 years, *n* = 9)	66.6%	33.3%	.39*
Elderly pregnancy (age ≥35 years, *n* = 19)	68.4%	31.6%	.35*
Gestational age at delivery (week)	39.0 ± 1.2	38.0 ± 1.3	.07
Low birth weight (baby birth weight <2500 g)	1.9%	35.0%	<.001
Number of delivery by Cesarean section (%)	33.3%	80.0%	<.001
Primigravida (*n* = 42, %)	69.0%	31%	.12**
Baseline serum creatinine (mg/dL ±SD)	0.52 ± 0.1	0.56 ± 0.1	.09

* *p* values compare to normal maternal-age pregnancy.

** *p* values compare to multiparity.

### Association between serum magnesium, ionized magnesium, ionized magnesium fraction and preeclampsia

**Figure 1.**
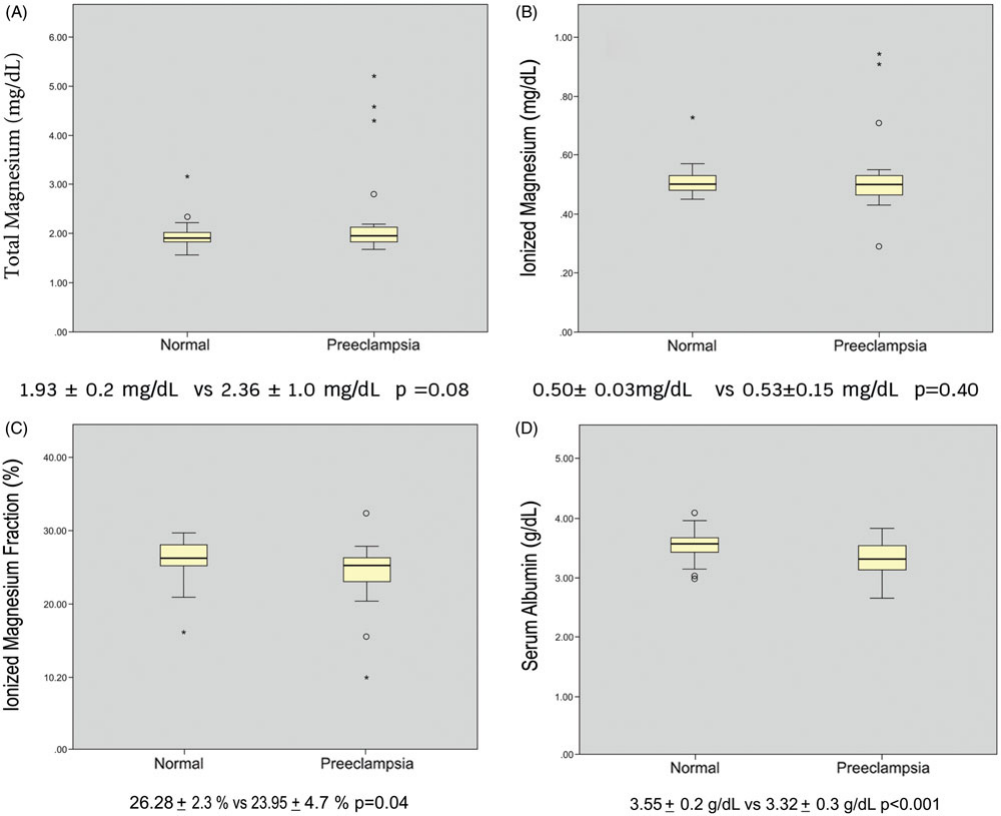
The box plots of the mean and SD of laboratory results: they demonstrated total magnesium levels (Picture A), ionized magnesium levels (Picture B), ionized magnesium fraction (%) (Picture C) and serum albumin levels (Picture D) during normal pregnancy and pregnancy with preeclampsia. Only ionized magnesium fraction and serum albumin levels showed the significant different between normal pregnancy and preeclampsia.

The Student’s *T*-test was employed to compare the levels of total magnesium, ionized magnesium, ionized magnesium fraction and serum albumin among women with normal pregnancy and preeclampsia. The ionized magnesium fraction and serum albumin levels of the normal pregnancy were significantly higher than those of the preeclampsia group (26.28 ± 2.3% vs. 23.95 ± 4.7% *p* = .04 and 3.55 ± 0.2 g/dL vs. 3.32 ± 0.3 g/dL, *p* < .001, respectively) (Figure 1). However, there was no significant difference in the total magnesium and ionized magnesium levels between the two groups (1.93 ± 0.2 mg/dL vs. 2.36 ± 1.0 mg/dL, *p* = .08 and 0.50 ± 0.03 mg/dL vs. 0.53 ± 0.15 mg/dL, *p* = .40, respectively). In addition, there was no statistical difference of the ionized magnesium fraction between the teenage and normal maternal age groups (23.48 ± 4.1% vs. 25.90 ± 3.0%, *p* = .26), and between the elderly and normal maternal age groups (25.91 ± 2.3% vs. 25.87 ± 3.2%, *p* = .98).

### The ionized magnesium fraction for prediction of preeclampsia

As mentioned earlier, factors which were found different between the normal pregnancy and preeclampsia groups included serum albumin levels and the ionized magnesium fraction. However, the low serum albumin levels may have resulted from nonspecific causes like illness or inflammation, and the change in serum albumin levels may have been a consequence of preeclampsia, not a cause. In addition, albumin is a binding protein of magnesium, change in albumin levels may affect the ionized magnesium fraction and interfere with results of logistic regression analysis. Therefore, we decided to use only the ionized magnesium fraction for an analysis to figure out risk factors of developing preeclampsia by logistic regression analysis.

**Figure 2.**
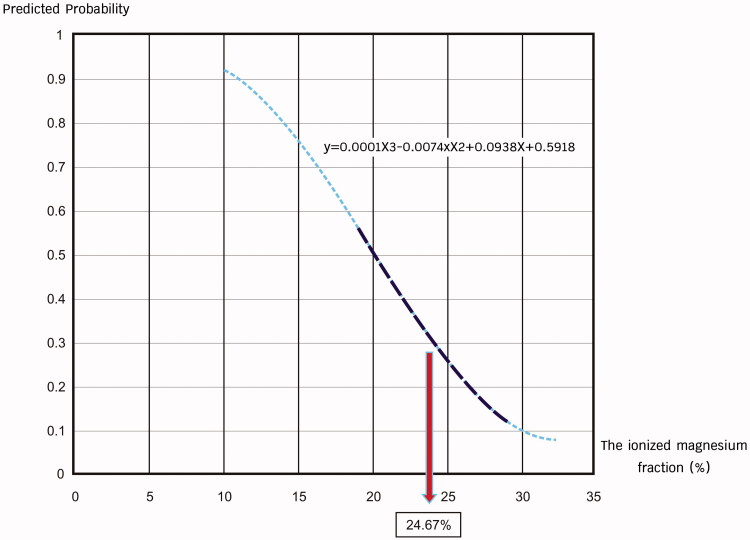
Curve of estimation on disease probability: the graph showed association between the probability of preeclampsia and the ionized magnesium fraction. The maximum slope indicated a cut point value of 24.67%, meaning that patients with ionized magnesium fractions of less than 24.67% were at higher risk of preeclampsia.

We incorporated the ionized magnesium fraction into a logistic regression analysis to estimate the probability of preeclampsia and obtained a curve of estimation (see Figure 2). The maximum slope of the curve suggested that the probability of disease maximally increased when the ionized magnesium fraction was <24.67%, which was used as a cut point for prediction. Then we categorized the participants into two groups, namely the ‘low ionized magnesium fraction’ group (ionized magnesium fraction <24.67%) and the ‘normal ionized magnesium fraction’ group (ionized magnesium fraction ≥24.67%).

We made a further logistic regression analysis to predict the probability of disease in the low ionized magnesium fraction group to test the hypothesis that the low fraction acted as a risk factor of preeclampsia. The results of logistic regression analysis using the ionized magnesium fraction in the form of a categorical variable provided a significant equation for the prediction of preeclampsia with an odds ratio of 4.41. In other words, the pregnant women with low ionized magnesium fraction had increased risk of preeclampsia by 4.41 times when compared to the normal ionized magnesium fraction group (unadjusted OR = 4.41, 95% CI: 1.46–13.40) as shown in Table 2.

**Table 2.** Association between preeclampsia and magnesium, teenage and elderly primigravida.

	Unadjusted OR (95% CI)	Adjusted OR* (95% CI)	*p* value (for adjust OR)
The ionized magnesium fraction	4.41 (1.46, 13.40)	5.55 (1.66, 18.64)	.006
(<24.67%)			
Teenage primigravida	5.47 (1.85, 35.42)	8.64 (1.179, 63.308)	.034
Elderly primigravida	11.11 (1.09, 113.78)	16.90 (1.48, 193.39)	.023

* The results of multivariate regression analysis also demonstrated the Ombinus *p* values = .001 with −2.021 of constant equation value. The coefficient values were 1.715, 2.157 and 2.827 for ionized magnesium fraction, teenage primigravida and elderly primigravida, respectively.

Therefore, the logistic equation = 1.715 (the ionized magnesium fraction) + 2.157(teenage primigravida) + 2.827(elderly primigravida) − 2.021.

### Ionized magnesium fraction-based equations incorporating clinical variables for prediction of preeclampsia

When taking only the maternal age into account for disease prediction, we found no significance of correlation between the age and preeclampsia as seen in Table 1. Nevertheless, we found more interesting results when incorporating additional factors concerning the first pregnancy (primigravida) into the categorization of participants in the logistic regression analysis. The results of logistic regression analysis are shown in Table 2. The results demonstrated that the ionized magnesium fraction, teenage primigravida and elderly primigravida could be risk factors of preeclampsia with a significant Omnibus *p* values of .001 for the overall equation and the *p* values of each variable also showed significant results as seen in Table 2.

Based on the results of logistic regression analysis, we created Equation (1). Then the goodness-of-fit test was used to assess model adequacy and the results suggested that expectations and perceptions were positively correlated with each other (*p* = .614), indicating that the model was applicable for disease prediction. Subsequently, we developed a predictive model for preeclampsia (Equation (2)).

(1) Logistic equation (Logit)=1.715 (the ionized magnesium fraction) +2.157 (Teenage primigravida) +2.827(Elderly primigravida) - 2.021

Since the probability of disease = [Exp(Logit)/1 + Exp(Logit)],

(2) A score predicting preeclampsia=Exp [(1.715×M)+(2.157×T)+(2.827×E)-2.021)] ÷{1+Exp [(1.715×M)+(2.157×T)+(2.827×E)-2.021)]}

*M* referred to the ionized magnesium fraction; *M* = 1 if an ionized magnesium fraction was <24.67% and *M* = 0 if an ionized magnesium fraction was ≥24.67%.

*T* referred to teenage primigravida, *T* = 1 if a patient was considered as teenage primigravida and *T* = 0 if a patient was not considered as teenage primigravida.

*E* referred to elderly primigravida, *E* = 1 if a patient was considered as elderly primigravida and *E* = 0 if a patient was not considered as elderly primigravida.

As the equations yielded from the logistic regression analysis were derived from the calculation of the probability of disease, the range of scores was 0–1.

### The accuracy of the ionized magnesium based equation model for predicting preeclampsia

**Figure 3.**
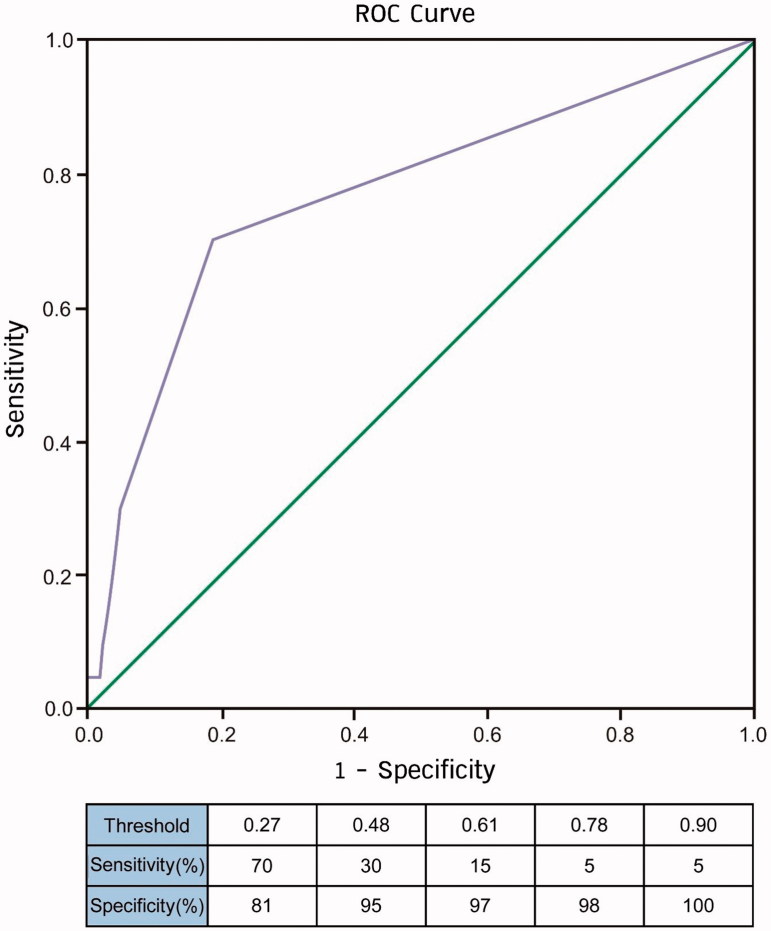
The ROC of the predictive accuracy of an ionized magnesium fraction-based equation model for preeclampsia: the ionized magnesium fraction-based equation model was derived from the logistic regression analysis on the fraction, teenage as well as elderly primigravida. The area under ROC curve was 0.77, indicating a significant degree of discrimination (*p* < .001). Scores of >0.27 were highly suggestive of preeclampsia with 70% sensitivity and 81% specificity.

We used the receiver operating characteristic (ROC) curve to evaluate accuracy of the ionized magnesium fraction-based equation model (teenage and elderly primigravida with low ionized magnesium fraction) as shown in Figure 3. The area under the curve was 0.77 with *p* < .001, which indicated a high degree of discrimination.

In addition, the ROC curve suggested that a score of >0.27 could serve as the best threshold for screening preeclampsia with 70% and 81% for sensitivity and specificity, respectively. Thus, a pregnant woman with a score of >0.27 would be at risk of preeclampsia and require close monitoring and constant medical attention.

## Discussion

As we know, preeclampsia has been a significant problem in clinical practice. It has been reported to increase mortality in mothers and children and associated with the necessity of delivery by Cesarean section, low birth weight, preterm labor and eclampsia [10–16]. Because of our small sample size; however, we did not find other complications like acute kidney injury, hepatitis or HELLP which could be also found in patients with preeclampsia.

Previous literature has demonstrated that magnesium levels may have decreased during pregnancy [4,8]. Nevertheless, we did not find the statistical significance in total magnesium and ionized magnesium levels between the normal pregnancy and the preeclampsia group, which indicated that total and ionized magnesium levels failed to serve as indicators of preeclampsia in our study. Changes in the total and ionized magnesium levels when used to calculate ionized magnesium fraction helped to develop equations suggesting that the ionized magnesium fraction acted as an indicator of preeclampsia. Our study pointed out that a pregnant woman who had an ionized magnesium fraction of less than 24.67% was at increased risk of preeclampsia during the second half of pregnancy. The results also indicated that the fraction was a more appropriate indicator in the prediction of disease.

Though decrease in albumin levels may increase ionized magnesium fraction, we found significant decrease in magnesium fraction and decrease in albumin concentrations. This may have suggested changes in other binding proteins in addition to albumin during pregnancy, mobilization of ionized magnesium into its complex form, or magnesium influx into cells, which may have played essential roles in pathophysiology of preeclampsia. Numerous reports have shown other approaches to the prediction of early preeclampsia, for example, Ghosh et al. reported that PIGF is a prominent and potential biomarker candidate. Additionally, in combination with uterine artery Doppler velocimetry, it can yield a substantially higher power of prediction [26,27]. Other predictors mentioned earlier also has its own advantages, however, all of them are advanced investigation which would require further expenditure. On the other hand, serum ionized magnesium is an available investigation in today’s general practice. Its discovery of prediction use will be exceedingly applicable worldwide. This study has shown that its level marks as a beneficial predictor rather than being just a factor for endothelial dysfunction. The further study of changes in binding protein of magnesium and monitoring of both biomarkers especially during the second half of pregnancy may assist healthcare professionals in preventing preeclampsia and providing knowledge for further research on pathophysiology of preeclampsia. In addition, the power of prediction to differentiate preeclampsia from other causes of hypertension related to pregnancy should be further investigate.

A multi-regression analysis indicated that the ionized magnesium fraction could assist in predicting occurrence of preeclampsia and it was used along with other clinical risk factors, such as teenage and elderly primigravida, in the development of the following ionized magnesium fraction-based equation so as to figure out a predictive score of disease.

A score predicting preeclampsia=Exp [(1.715×M)+(2.157×T)+(2.827×E)-2.021)]÷{1+Exp [(1.715×M)+(2.157×T)+(2.827×E)- 2.021)]}

Based on the simplified and practical equation, a pregnant woman who had a score of more than 0.27 had increased risk of preeclampsia with 70% sensitivity and 81% specificity. The ROC analysis pointed out that a healthcare professional team needed to monitor total and ionized magnesium levels in addition to routine laboratory testing of an antenatal care program.

We also suggested that the further studies of early at 20 weeks of pregnancy in order to accurately predict the risk of preeclampsia would be considered. This would be beneficial for the decision to correct the ionized magnesium fraction during the second half of pregnancy with an attempt to prevent preeclampsia and complications. There also should be research on whether the correction of serum ionized magnesium fraction alters magnesium levels in other fluid compartments if magnesium is administered in the patient without history of hypertension.

Preeclampsia is still a disease of concern in clinical practice. Measurement of ionized magnesium fraction and use of magnesium fraction-based equations enable healthcare professionals to provide better patient care, especially during the second half of pregnancy. Such approach can be substantially advantageous for informing patients and making management decisions.
